# Impact of Single-Family Room Care on Neonatal Outcomes: A Systematic Review

**DOI:** 10.7759/cureus.88499

**Published:** 2025-07-22

**Authors:** Khaled El-Atawi, Jubara Alallah, Mansour Al Qurashi, Maysa Saleh

**Affiliations:** 1 Pediatrics/Neonatal Intensive Care Unit, Latifa Women and Children Hospital, Dubai, ARE; 2 Pediatrics/Neonatology, King Saud Bin Abdulaziz University for Health Sciences, Jeddah, SAU; 3 Pediatrics and Child Health, Al Jalila Children's Hospital, Dubai, ARE

**Keywords:** individualized care, neonatal intensive care unit (nicu), neurodevelopmental outcomes, parent-infant interactions, single-family room

## Abstract

The primary aim of the present study is to examine the literature regarding the advantages and disadvantages of single-family room (SFR) care for neonatal outcomes. The researchers used the systematic review approach, where several databases were used, including PubMed, Medical Literature Analysis and Retrieval System Online (MEDLINE), Cochrane, and Scopus, using Preferred Reporting Items for Systematic Reviews and Meta-Analyses (PRISMA) as a methodological path to identify, screen, and select the studies that were included in the current research and analyzed to extract the information needed for the achievement of the research objectives. The results revealed that neonatal intensive care units (NICUs) can be improved structurally and operationally, which may lead to better outcomes for premature infants and their families. One promising strategy is to provide SFRs within the NICU. SFRs increase the chances for a neonate and their family to have uninterrupted time together.

## Introduction and background

The single-family room (SFR) design is being used more and more in neonatal intensive care units (NICUs) because of the notable advantages over the conventional unit-based structure in terms of both short-term and long-term health and neurodevelopmental outcomes [1, 2]. Increased knowledge of the developmental requirements of premature infants and the emphasis on creating environments that encourage and facilitate family participation [3].

Every SFR has all the necessary supplies and equipment for both maternity and newborn care [1]. Parents are allowed to stay with their newborns all the time in the SFRs. The coordination between parents and medical professionals improves information sharing and collaborative decision-making [1]. Families may benefit from SFRs because they offer a private and secure environment to strike a balance between their needs for socialization and privacy [4].

When compared to parents of infants admitted to open bay unit (OBU) NICUs, parents of preterm infants admitted to SFR NICUs had better results. It was discovered that parents of preterm infants admitted to SFRs had higher levels of parental presence, involvement, skin-to-skin care, empowerment, degree of family-centered care (FCC), and satisfaction, as well as lower NICU-related stress levels upon discharge [5].

It is widely acknowledged that they improve parental comfort and privacy, lower the risk of nosocomial infection, and enable customized and developmentally appropriate levels of sensory input for infants. However, there are some possible issues with using SFRs exclusively. The primary one is the potential harm that a preterm neonate could experience from an environment lacking critical sensory inputs. Feelings of loneliness can also have a detrimental effect on family members and employees [6].

Statement of the problem

Despite improvements in neonatal care, preterm and critically ill infants continue to be at high risk of adverse health outcomes such as infections, developmental delays, and long hospital stays. Traditionally, open-air NICUs were criticized for their negative effects on infant performance, including increased exposure to noise, decreased parental involvement, and diminished privacy.

One novel alternative idea that aims to foster a more individualized and family-oriented setting is SFR care, which enhances clinical results, fosters the practice of developmental care, and fortifies family bonds. Research on how SFR affects birth outcomes is still inconclusive, despite these clear advantages. Therefore, the current study seeks to determine whether SFR care offers any noteworthy benefits over conventional ward-style care.

Questions of the study

The current research is formulated from a methodological standpoint to help answer two integral research questions: What is the positive impact of SFR care on neonatal outcomes? And what is the negative impact of SFR care on neonatal outcomes?

## Review

Specifications of SFRs

Due to the increasing number of premature births, prematurity is turning into a serious public health concern [7]. In the NICU, preterm birth is one of the main reasons for admission [8]. One important solution to enhance care and lower developmental morbidity in preterm infants is the SFR [9]. In its most basic form, the NICU is a microsystem created to care for premature or ill newborns [10].

Without a doubt, the functional organization and physical layout can either improve or worsen the capacity to deliver high-quality care. Infants, families, and staff should all have their medical, developmental, educational, emotional, and social needs met in the NICU [6]. The single-family architecture resulted in greater involvement, less interference, and closer proximity between parents and infants [11, 12]. 

The implementation of SFR units in NICUs has been associated with numerous favorable consequences for newborns, their families, and the medical staff, as observed in multiple studies conducted over the past two decades [2].

Many people agree that SFRs are crucial to the layout of a contemporary NICU. It is widely acknowledged that they improve parental comfort and privacy, lower the risk of nosocomial infections, and enable tailored and developmentally appropriate levels of sensory input for infants [6]. The advantages can be discussed at more than one level as follows:

Even though the mortality rate for high-risk infants has decreased due to advancements in neonatology. Evidence supporting the advantages of FCC in NICUs is growing. The FCC is founded on the understanding that, despite their illness or early birth, newborns have the right to be near their parents as human beings [13].

Advantages of using SFRs as accommodation rooms

Compared to neonates in OBUs, those in SFRs have shorter stays and greater weight gain [14]. Apart from more effective weight gain, babies born and nurtured in SFRs demonstrated better neurodevelopment. Controlling noise levels and light exposure were environmental elements that also helped to get these better results [15].

In SFRs, infections picked up in hospitals are less frequent among babies. Jansen et al. (2022) [16] confirm that infants in the SFR NICU behaved better in terms of attention, physiologic stress, hypertonicity, lethargy, and pain; needed fewer medical procedures; had a shorter gestational age at full enteral feed; and experienced less sepsis [16, 9]. Late-onset sepsis is less probable in preterm infants housed in SFR receiving family-integrated care (FIC). Because exclusive breastfeeding is more common and sepsis is less common, SFRs are advised when hospitalizing preterm infants [5].

According to Jones et al. (2023) [17], the SFR model improves the family-centeredness of care. It makes family engagement easier and increases parents' confidence in early childhood care [18, 5]. Soleimani et al. (2020) [15] also showed that SFR care encouraged a more family-centered approach that improved patient confidentiality and parent involvement, which in turn improved parents' contentment and participation in caregiving duties [15]. Parent participation was further defined as follows: skin-to-skin care, involvement, and presence [5].

Increasing parent-infant interactions in an SFR in the NICU may help preterm infants' language development. Recognizing parents as primary carers and partners in a cooperative setting, family-based care can integrate these support strategies [19]. In SFRs, mothers had stronger emotional ties to their children, more frequent caretaking activities, and more physical contact with them [5]. Room design also affects the physical bond and ability to breastfeed between the mother and her child [18].

When a premature baby is admitted to the NICU after birth, many of the parents experience depression and symptoms of stress. Compared to mothers in the open ward, mothers in the SFR were less stressed and anxious [20, 21]. It encourages privacy, which gives the baby more time and attention, strengthening the bond and allowing for more individualized care [22, 16].

Disadvantages of the SFR model

Despite the SFR’s advantages, the following disadvantages can be outlined as well upon implementing it: The SFR model may increase parents' sense of social isolation, particularly in situations where there are no other families in the NICU [5]. After losing the option that OBUs provided, parents expressed dissatisfaction over the lack of interaction with other families [16]. Apart from the primary consequences, there are other problems to consider, such as challenges in effective communication between families and doctors [17]. Teixeira-Poit et al. (2023) [22] show that compared to open-bay models, the staff felt more alone and struggled to coordinate and communicate. Access and service issues were also brought about by the redesign, including lengthy travel times for staff members between areas and logistical issues with the medical inventory [22]. Additionally, some parents expressed that they felt lonely and that they would have enjoyed the company that OBUs offered [22].

Methodology

A number of databases were used, including PubMed, Medical Literature Analysis and Retrieval System Online (MEDLINE), Cochrane, and Scopus, using Preferred Reporting Items for Systematic Reviews and Meta-Analyses (PRISMA) as a methodological path to identify, screen, and select the studies that were included in the research and analyzed to extract the information needed to achieve the research objectives.

To carry out the current study on the effect of SFR care on newborns, PubMed, MEDLINE, Cochrane, and Scopus were searched systematically. A non-random study was conducted by applying the PRISMA recommendations to ensure the study was structured properly. The used keywords were "NICU," "SFR," "single room," "neonatal," "neonatal intensive care," "neonatal consequences," "single-family unit," "private room," and their derivatives. Relevant information was retrieved in greater numbers by applying both Boolean operations and syntax matching to different databases.

Date ranges and language limits were applied depending on each database’s capabilities, though the exact details were not made public. Clear guidelines were established for selecting studies based on specific inclusion criteria. Research that aligned with the overarching aims focused on neonatal patients who had received SFR care in the NICU, with those in OBUs serving as the comparison group.

In this context, assistive tools proved beneficial in conducting procedures within the PRISMA flowchart. For instance, the updated 2020 version of PRISMA 1.0 was used. Additionally, Zotero software (Corporation for Digital Scholarship, Vienna, VA) was employed to collate, organize, and identify duplicate studies within a designated library. Moreover, ASReview LAB, developed by Utrecht University (Utrecht, Netherlands), was used for the title-and-abstract screening process through a Python package utilizing TF-IDF, Naïve Bayes, maximum query strategy, and dynamic resampling algorithms and models.

Subsequently, the screened studies were considered subject to further screening to detect the ones that are eligible for being evaluated in accordance with the following inclusion and exclusion criteria (Table 1).

**Table 1:** Inclusion and exclusion criteria PDF: portable document format

Inclusion criteria	Exclusion criteria
Studies published between 2020 and 2025	Studies published before 2020
Studies available in full format	Studies available in abstract versions only
Studies written in English	Studies written in any language other than English
Studies that are relevant to the research question	Studies that drift away from the main variables within the research question
Studies that provide experimental, quantitative, or clinical-trial research findings	Studies that are conducted as review papers, book chapters, or theoretical insights on reports and/or briefs
Studies available in PDF format	Studies that are not available in PDF format

The final assortment of studies was selected for inclusion in the data analysis phase, during which each study was reviewed to extract its aims and objectives, methodologies, findings, and research gaps. This information was compiled in a Microsoft Excel spreadsheet (Microsoft Corp., Redmond, WA). Lastly, the extracted data were used to identify the impact of SFR care on neonatal outcomes.

Data analysis procedure

**Figure 1:**
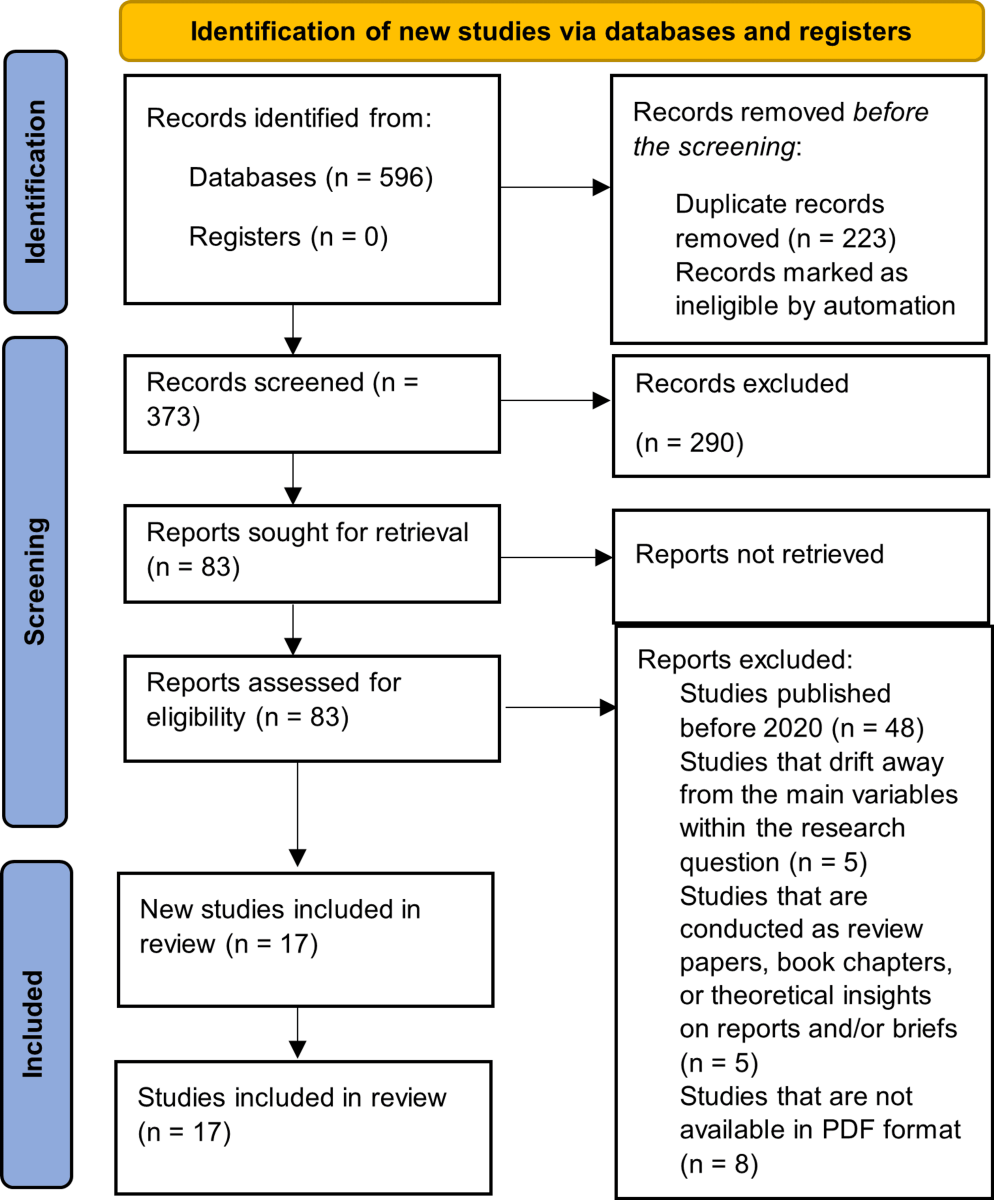
A PRISMA flowchart outlining the study selection process PRISMA: Preferred Reporting Items for Systematic Reviews and Meta-Analyses; PDF: portable document format

Following the PRISMA flowchart, the first phase pertained to the identification procedure, where the previously formulated search strands were used in each of the previously determined databases. Accordingly, upon using the search keywords and strands, a total of 151, 128, 138, and 179 results were identified on PubMed, MEDLINE, Cochrane, and Scopus, respectively. These studies amounted to 596 in total and were then uploaded to Zotero to help organize them and find the duplicates to merge them and move into the next phase (Figure 1).

Subsequently, Zotero found 223 studies that were considered duplicates, and they were merged to bring down the number of results to 373 studies. As a result, the screening process commenced by uploading all 373 studies to ASReview LAB, an automated screening tool, to help screen the eligible studies for being subject to the verification phase by using the previously outlined inclusion and exclusion criteria.

In this phase, the algorithms were taught within the machine-learning system in the ASReview LAB to mimic the same mechanism used to deem studies relevant or irrelevant, which selects only those studies that include relevant keywords in the title and/or abstract section, directly or indirectly, corresponding with the subject at hand. Then, the machine was ready to implement the same mechanism and recommend studies to be selected or excluded, which it did by making sure that it included topics that correspond with the need to investigate the impact of SFR care on neonatal outcomes.

Consequently, applying the automated title-and-screening method yielded 83 relevant studies that were deemed eligible for being evaluated using the inclusion and exclusion criteria, while 290 studies were considered irrelevant. Moreover, the 83 studies were uploaded once again to Zotero to organize them, find the duplicates, and review their eligibility criteria in order to select the included studies for the final analysis phase. Additionally, a CSV file was created that included all 83 studies to help evaluate them using these criteria. Consequently, the following studies were included/excluded for the following reasons (Table 2).

**Table 2:** Number of included studies upon the application of the exclusion criteria on eligible studies PDF: portable document format

Exclusion Criteria	N
Studies published before 2020	48
Studies available in abstract versions only	-
Studies written in any other language than English	-
Studies that drift away from the main variables within the research question	5
Studies that are conducted as review papers, book chapters, or theoretical insights on reports and/or briefs	5
Studies that are not available in PDF format	8
Total	66

Eventually, the final set of studies that were determined upon the application of the inclusion and exclusion criteria amounted to 17 studies that met all the inclusion criteria and were deemed eligible for the analysis phase. The following table analyzes the studies in terms of their aims, methodologies, and findings (Table 3).

**Table 3:** Analytical review of the included studies SFR: single-family room; NICU: neonatal intensive care unit; SSC: skin-to-skin contact; FCC: family-centered care; LOS: late-onset sepsis; FIC: family integrated care; OB: open bay; db: decibel

Study	Aim	Methodology	Key findings
Machry et al. (2023) [23]	Explore how SFR design supports family engagement	Observations, interviews, grounded theory	Design features (e.g., private bathrooms) enhance family engagement
Lehtonen et al. (2020) [12]	Role of SFR interior design in family engagement	Observations, interviews at two NICUs	SFRs enhance collaborative caregiving through thoughtful design
Campbell et al. (2021) [21]	Assess the SFR effect on parental presence and well-being	Cohort study with diaries and surveys	SFRs increased presence; no improvement in maternal well-being
Kainiemi et al. (2021) [11]	Evaluate SFR's impact on SSC and family-centered care	Pre/post-cohort comparison	More parental time in SFRs; no change in SSC or perceived FCC
Pellikka et al. (2020) [24]	Explore parents’ roles in SFR NICU	Qualitative interviews with parents	Parents felt more responsible; they need nursing support and clear guidelines
Larsen et al. (2024) [4]	Emotional impact of SFRs on nurses	Observations and interviews	SFRs increased emotional load; bonds with parents helped mitigate stress
Soni et al. (2021) [25]	Staff perceptions of SFR NICUs	Survey of NICU staff	SFRs improved privacy but raised concerns about isolation and safety
Van Veenendaal et al. (2020) [5]	Assess SFRs’ effect on LOS and neonatal outcomes	Before-and-after study with mediation analysis	Reduced LOS and hospital stay; effect mediated by reduced PN use
Doede et al. (2020) [26]	Evaluate the impact of infant-parent rooms on outcomes	Survey + cohort study across NICUs	Reduced mortality, morbidity, and LOS in SFR-equipped units
Puumala et al. (2020) [14]	Compare outcomes pre- and post-SFR transition	Retrospective analysis of NICU encounters	Lower LOS in preterms; increased LOS and sepsis in term/post-term infants
Parm et al. (2023) [27]	Microbial effects of early FIC	Prospective cohort with microbial testing	Lower hospital-acquired strains, greater diversity, and earlier SSC
Grundt et al. (2021) [18]	Assess milk production and breastfeeding in SFR vs. OB	Longitudinal observational study	SFRs led to earlier initiation, more exclusive breastfeeding
Lyngstad et al. (2022) [28]	Improve pain management and parental involvement	Quality improvement project	Improved guideline compliance and parental participation
Capriolo et al. (2022) [29]	Study NICU sound exposure	dB measurement with app; statistical analysis	SFRs had lower peak noise levels but still exceeded safe limits
Silva et al. (2024) [30]	Evaluate stress and regulation in SFR vs. OB infants	Observational study of 40 neonates	SFR neonates showed fewer stress behaviors, better self-regulation
Dadiz et al. (2020) [31]	Identify safety threats pre-/post-SFR NICU move.	Simulation and thematic analysis	305 threats identified; 91% resolved through pre-occupancy simulation
Dadiz et al. (2023) [32]	Review the simulation in safe NICU transition	In-situ simulations and operations testing	Simulation identified design flaws and improved NICU transition safety

Findings

NICUs can be improved structurally and operationally, which may lead to better outcomes for premature infants and their families. One promising strategy is to provide SFRs within the NICU. The SFR increases the chances for a neonate and their family to have uninterrupted time together. More time spent together can help establish a bond between the baby and family members, which is essential when that family has faced so many challenges, given the fact that when a neonate is born prematurely, the chances of bonding are drastically reduced. Nevertheless, the authors of the previously reviewed studies reported certain advantageous impacts of SFRs.

Firstly, the use of FIC in SFRs has helped reduce the number of hospital-acquired strains. This came about because SFRs allowed for the earlier direct contact that preterm infants need, as well as allowing more mothers to use natural breastfeeding as an option for meeting their neonates’ nutritional needs. Secondly, some SFRs had a negative impact on outcomes. This means that preterm neonates received better care than their term and post-term counterparts. Part of this seems to relate to the preterm infant's increased need for individualized care, and the noise control is part of that.

Lastly, SFR care had some conflicting impact on nursing staff, whose attitudes presented mixed feelings. Though they liked the SFRs for the sheer fact that they provided increased privacy and improved lines of communication, they were also subject to immense levels of pressure, emotional stress, and constant tendencies to further improve their skills to meet the needs of the entire family. This concludes that SFRs can have both positive and negative impacts on neonates, their families, and even the nursing staff in general.

Discussion

Upon reviewing and analyzing the previously extracted studies, the following conclusive reflection and discussion of the findings of such studies can be outlined.

Impact of SFR Specification on Family-Oriented Care

SFR care in NICUs research highlights diverse impacts on family, neonatal outcomes, and staff experiences. A significant focus area in many studies is the FCC influenced by SFR design. Machry et al. (2023) and Lehtonen et al. (2020) [23,12] emphasize the way specific design elements, such as private toilets, storage rooms, and family zones, lead to increased levels of family engagement so that parents may become more active in infant care. This aligns with research by Campbell et al. (2021) and Kainiemi et al. (2021), where SFRs were related to increased parental presence [21,11].

Though SFRs allowed greater parental involvement, Campbell et al. (2021) [3] found no improvement in maternal well-being, and Kainiemi et al. (2021) [11] found no increase in skin-to-skin contact [11]. Such conflicting results suggest that, while SFR room designs have structural potential for family involvement, their impact on caregiving and emotional contact is uncertain. Neonatal health outcomes find benefits and risks in SFRs. Studies by Van Veenendaal et al. (2020) [5] and Doede et al. (2020) [26] demonstrated SFRs to be associated with reduced incidence of late-onset sepsis (LOS) and hospitalization, as well as overall mortality and morbidity rates, confirming the hypothesis that family-centered care improves the health of preterm babies [5, 26].

Varying Impact of Using SFRs

In contrast, Puumala et al. (2020) introduced a more refined representation, with lower LOS in preterm infants but higher LOS and sepsis rate in term/post-term infants during SFR transition [14]. Such contradictory findings stress that more subtle measures are needed and suggest that SFR care is not consistently positive across all neonatal subgroups. Similarly, Parm et al. (2023) [27] mentioned that SFRs with early FIC lowered hospital-acquired strains and enriched microbial diversity, showing the microbiological advantage of parent participation [27].

These advantages are greatly dependent on the organizational and operational provision of FIC. Determinants of the environment and staff perceptions also limit the impact of SFR care. Capriolo et al. (2022) [29] indicated SFRs were better at maintaining noise control compared to open-bay NICUs, yet both units continued to surpass noise recommendations, which impacted neonatal development and stress [27]. Staff attitudes, researched by Soni et al. (2021) and Larsen et al. (2024), identified that while SFRs enhanced privacy and noise reduction better, these also promoted isolation of staff, emotional workload, and patient safety [25, 4]. Larsen et al. (2024) emphasized the way continuous parental visibility in SFRs placed undue emotional load on nurses, despite effective nurse-parent relationships lessening the impact at times [4].

Solutions Towards Maximizing SFRs’ Benefits and Minimizing Their Negative Impact

Pellikka et al. (2020) [24] noted that parents in SFRs felt more responsibility for the care of their infant, and there was a greater need for more rigid rules in order to reconcile parent autonomy and professional action. Simulation research has helped significantly counteract problems related to safety and operation in SFR NICUs [24]. Dadiz et al. (2020) and Dadiz et al. (2023) both emphasized simulation-based approaches as imperative in identifying latent safety threats (LSTs) in NICU transfers [31, 32]. The study showed how in-situ simulation and pre-occupancy testing were effective in pinpointing possible peril in communication, system breakdown, and design susceptibility, leading to improved safety before full integration of patients. This preventive approach is contrasted with reactive modifications observed in studies like those of Soni et al. (2021) [25], whose personnel problems were preceded by workflow adaptation post transition.

It should also be noted that the current research was not registered in the International Prospective Register of Systematic Reviews (PROSPERO) or any other reputable database or internationally renowned systematic review registry across the globe, for that matter. This decision was made due to the tight schedule and inapt timeline of the research, which did not cohere with the approach of the registry phases. Nevertheless, the systematic review followed the steps of the PRISMA flowchart rigorously. 

## Conclusions

The end assessment of the study proves that SFR care for NICUs holds substantial merits in improving neonatal and parent outcomes, particularly in preterm birth. SFRs advance FCC through parental engagement, skin contact, and accommodation for individualized, developmentally supportive environments. Numerous studies have repeatedly shown that babies in SFRs experience lower stress, fewer hospital-acquired infections, decreased length of stay, and better neurodevelopmental outcomes. Furthermore, families gain too from greater privacy, higher bonding, and satisfaction. The SFR design also encourages practices like exclusive breastfeeding and early language stimulation that benefit infants' physiological and emotional development.

However, the study also emphasizes some limitations and inconsistencies in the outcomes of SFR care. While architectural and operational features of SFRs are advantageous, their success hinges on adequate staff, efficient communication systems, and implementation of family-centered protocols. Certain studies address staff emotional burden due to isolation and overpresence by parents, but others suggest that SFRs may not necessarily be equally helpful for all subgroups of neonates; term infants, e.g., may not be as helped as compared to preterm infants. Issues regarding parental solitude and reduced peer support in contrast to OBUs are also brought forth. These results imply that although SFR care holds transformative potential, its adoption should be context-dependent and buttressed by far-reaching organizational initiatives in order to ensure its fullest possible impact across heterogeneous NICU environments.

## References

[1] Stelwagen MA (2023). Empowerment of parents in neonatal level 2 care: overcomming dilemmas and embracing chances. https://research.vu.nl/en/publications/empowerment-of-parents-in-neonatal-level-2-care-overcomming-dilem.

[2] Alsaleem M (2019). Single-family room, neonatal intensive care unit design. Biomed J Sci Tech Res.

[3] White RD (2010). Single-family room design in the neonatal intensive care unit-challenges and opportunities. Newborn Infant Nurs Rev.

[4] Larsen JN, Hansson H, Beck SA, Zoffman B (2024). Single-family rooms in neonatal intensive care: a qualitative analysis of fathers', mothers' and nurses' experiences. J Neonatal Nurs.

[5] van Veenendaal NR, van der Schoor SR, Heideman WH (2020). Family integrated care in single family rooms for preterm infants and late-onset sepsis: a retrospective study and mediation analysis. Pediatr Res.

[6] Dunn MS, MacMillan-York E., Robson K (2016). Single family rooms for the NICU: pros, cons and the way forward. Newborn Infant Nurs Rev.

[7] Séassau A, Munos P, Gire C, Tosello B, Carchon I (2023). Neonatal care unit interventions on preterm development. Children (Basel).

[8] Sharma Y, Pathak OK, Poudel B, Sharma A, Sapkota RP, Devkota K (2023). Preterm neonates admitted in the neonatal intensive care unit at a tertiary care centre: a descriptive cross-sectional study. JNMA J Nepal Med Assoc.

[9] Lester BM, Hawes K, Abar B (2014). Single-family room care and neurobehavioral and medical outcomes in preterm infants. Pediatrics.

[10] Smith C, Campbell D (2023). Neuroprotective Care of the NICU Infant Clinical Pathway. http://www.hopkinsmedicine.org/-/media/files/allchildrens/clinical-pathways/neuroprotective-care-of-the-nicu-infant-11_22_23.pdf.

[11] Kainiemi E, Hongisto P, Lehtonen L, Pape B, Axelin A (2021). Effects of single family room architecture on parent-infant closeness and family centered care in neonatal environments-a single-center pre-post study. J Perinatol.

[12] Lehtonen L, Lee SK, Kusuda S (2020). Family rooms in neonatal intensive care units and neonatal outcomes: an international survey and linked cohort study. J Pediatr.

[13] Lee J (2024). Neonatal family-centered care: evidence and practice models. Clin Exp Pediatr.

[14] Puumala SE, Rich RK, Roy L, Reynolds R, Jimenez FE, Opollo JG, Brittin J (2020). Single-family room neonatal intensive care unit design: do patient outcomes actually change?. J Perinatol.

[15] Soleimani F, Rostami FF, Nouri JM, Hatamizadeh N, Sajedi F, Norouzi M (2020). Impacts of the design of a neonatal intensive care unit (single-family room care and open-ward care) on clinical and environmental outcomes. Crescent J Med Biol Sci.

[16] Jansen S, Berkhout RJ, Te Pas AB (2022). Comparison of neonatal morbidity and mortality between single-room and open-bay care: a retrospective cohort study. Arch Dis Child Fetal Neonatal Ed.

[17] Jones CW, Moya F, Lynch N (2023). Unintended consequences of the neonatal intensive care unit environment: Integrative review of single-family room unit design. Adv Neonatal Care.

[18] Grundt H, Tandberg BS, Flacking R, Drageset J, Moen A (2021). Associations between single-family room care and breastfeeding rates in preterm infants. J Hum Lact.

[19] Heo JS, Kim EK (2024). Strategies to support language development in neonatal intensive care unit: a narrative review. Clin Exp Pediatr.

[20] Feeley N, Robins S, Genest C, Stremler R, Zelkowitz P, Charbonneau L (2020). A comparative study of mothers of infants hospitalized in an open ward neonatal intensive care unit and a combined pod and single-family room design. BMC Pediatr.

[21] Campbell-Yeo M, Kim T, Disher T (2021). Do single-family rooms increase parental presence, involvement, and maternal well-being in neonatal intensive care?. J Perinat Neonatal Nurs.

[22] Teixeira Poit S, Fields B, Kendrik F (2022). Teixeira-Poit receives NIH grant to study impact of neonatal intensive care unit design. https://www.ncat.edu/news/2022/09/nicu-nih-grant-study.php.

[23] Machry H, Joseph A, White R, Allison D (2023). Designing for family engagement in neonatal ICUS: how is the interior design of single-family rooms supporting family behaviors, from passive to active?. HERD.

[24] Pellikka HK, Pölkki T, Sankilampi U, Kangasniemi M (2020). Finnish parents' responsibilities for their infant's care when they stayed in a single family room in a neonatal intensive care unit. J Pediatr Nurs.

[25] Soni R, Fairhurst N, El Anbari M, Leslie A, Tscherning Wel-Wel C (2022). Staff perceptions and challenges of the single-family room design-experience of a greenfield level4 neonatal intensive care unit in the Middle East. Acta Paediatr.

[26] Doede M, Trinkoff AM (2020). Emotional work of neonatal nurses in a single-family room NICU. J Obstet Gynecol Neonatal Nurs.

[27] Parm Ü, Tiit-Vesingi A, Soeorg H (2023). Effect of early directed implementation of family-integrated care measures on colonisation with Enterobacteriaceae in preterm neonates in NICU. BMJ Paediatr Open.

[28] Lyngstad LT, Steinnes S, Le Marechal F (2022). Improving pain management in a neonatal intensive care unit with single-family room-a quality improvement project. Paediatr Neonatal Pain.

[29] Capriolo C, Viscardi RM, Broderick KA, Nassebeh S, Kochan M, Solanki NS, Leung JC (2022). Assessment of neonatal intensive care unit sound exposure using a smartphone application. Am J Perinatol.

[30] Silva NF, Linhares MB, Gaspardo CM (2024). Stress and self-regulation behaviors in preterm neonates hospitalized at open-bay and single-family room neonatal intensive care unit. Infant Behav Dev.

[31] Dadiz R, Bender J, Robin B (2023). Simulation-based operations testing in new neonatal healthcare environments. Semin Perinatol.

[32] Dadiz R, Riccio J, Brown K, Emrich P, Robin B, Bender J (2020). Qualitative analysis of latent safety threats uncovered by in situ simulation-based operations testing before moving into a single-family-room neonatal intensive care unit. J Perinatol.

